# Impact of *Parabacteroides distasonis* colonization on host microbiome, metabolome, immunity, and diabetes onset

**DOI:** 10.1530/JME-25-0025

**Published:** 2025-08-28

**Authors:** Khyati Girdhar, Audrey Randall, Keiichiro Mine, Clarissa Howard, Alessandro Pezzella, Dogus Dogru, Lukas Rhodes, Brady James, Umesh K Gautam, Dagmar Šrůtková, Tomas Hudcovic, Juan J Aristizabal-Henao, Michael Kiebish, Emrah Altindis

**Affiliations:** ^1^ Boston College Biology Department, Chestnut Hill, Massachusetts, USA; ^2^ Medical College of Wisconsin, Department of Pediatrics, Division of Endocrinology, McGee Diabetes Research Center, Milwaukee, Wisconsin, USA; ^3^ Laboratory of Gnotobiology, Institute of Microbiology of the Czech Academy of Sciences, Novy Hradek, Czech Republic; ^4^ BPGbio, Inc., Framingham, Massachusetts, USA

**Keywords:** Parabacteroides distasonis, type 1 diabetes, molecular mimicry, microbiome, autoimmunity

## Abstract

Type 1 diabetes (T1D) is caused by autoimmune destruction of pancreatic β-cells. The insulin B-chain 9–23 (insB9–23) peptide is a critical epitope in triggering T1D. In our previous study, we showed that *Parabacteroides distasonis*, a human gut commensal, contains an insB9–23 mimic in its hprt protein (residues 4–18). This mimic (hprt4-18) peptide activates insB9–23-specific T cells, and *P. distasonis* colonization enhanced diabetes in NOD mice. However, the impact of the *P. distasonis* colonization on inflammation, gut microbiome, intestinal immune cells, gut permeability, cytokine, and serum metabolome profiles remained unknown. Here, we investigated these effects using specific pathogen-free (SPF) and germ-free (GF) female NOD mice. *P. distasonis* colonization minimally impacted gut microbiome composition, altering only 28 ASVs. In *P. distasonis*-colonized mice, there was a reduction in T-helper, T-effector, and B-cell populations in the intraepithelial lymphocytes, indicating a potential decrease in immune activation. Furthermore, *P. distasonis* colonization did not alter serum metabolome and circulating cytokine profiles (except for a decrease in IL-15) and gut permeability gene expressions. *P. distasonis* colonization in GF NOD mice induced severe insulitis without affecting gut permeability. Interestingly, mice gavaged with heat-inactivated (HI) *P. distasonis* did not affect insulitis scores or immune cell composition. These findings support our hypothesis that *P. distasonis* functions as a gut commensal, exerting no effect on the gut microbiome, metabolome, gut permeability, intestinal immune cell composition, or nonspecific immune activation. Instead, *P. distasonis* appears to trigger an insB9–23-specific immune response, potentially accelerating T1D onset in NOD mice through molecular mimicry.

## Introduction

Type 1 diabetes (T1D) is an autoimmune disease characterized by the selective destruction of pancreatic β-cells by autoreactive T cells (Pugliese 2017). The incidence of T1D in children is rising annually, with an increase of 3.4% in Europe and 1.4% in the USA (Patterson *et al.* 2019). Genome-wide association studies have identified over 60 loci that influence the risk of developing T1D (Pociot 2017); however, genetics alone cannot account for the increasing incidence rates. Various environmental factors, including diet, birth mode, infections, and antibiotics, have also been studied (Christen *et al.* 2010, Op de Beeck & Eizirik 2016, Rewers & Ludvigsson 2016). However, the trigger of T1D autoimmunity remains elusive.

Over the past 20 years, the role of the gut microbiota in health and disease has become increasingly understood. The environmental factors mentioned above can induce functional and compositional changes in the gut microbiota (Rewers *et al.* 2018). Several studies have highlighted the continuous crosstalk between the immune system and gut microbes starting immediately after birth (Maynard *et al.* 2012). Microbial colonization and exposure to self- and non-self-antigens shape the host immune system early in life (Korpela *et al.* 2020). This coincides with the period when the incidence of T1D is most common. Previous reports have documented changes in gut microbiome composition and diversity in individuals with T1D (Dedrick *et al.* 2020). The DIABIMMUNE study, a longitudinal examination of the fecal microbiome in HLA- and age-matched infants, revealed that there was a higher abundance of pathobionts such as *Ruminococcus gnavus* in seroconverted children compared to seronegative subjects (Kostic *et al.* 2015). In a follow-up study, they found a higher prevalence of *Bifidobacterium* in Russians and LPS-producing *Bacteroides* species in Finns and Estonians. Interestingly, *Bacteroides* LPS inhibited innate immune signaling and endotoxin tolerance, and *Bacteroides dorei* LPS did not reduce autoimmune diabetes incidence in non-obese diabetic (NOD) mice (Vatanen *et al.* 2016). Strain-level analysis also revealed significant differences for *Bifidobacterium species* (Vatanen *et al.* 2019).

The Environmental Determinants of Diabetes in the Young (TEDDY) study, another key longitudinal cohort, used samples from 903 high-risk infants and reported *Parabacteroides* as the only genus significantly associated with T1D onset (Stewart *et al.* 2018). Similarly, the Innovative Approaches to Understanding and Arresting Type 1 Diabetes (INNODIA) study (Vatanen *et al.* 2024) identified *Parabacteroides distasonis* as one of the 30 most abundant species in newly diagnosed individuals (Vatanen *et al.* 2024). These longitudinal studies offer valuable insights into gut microbiome changes, identifying specific alterations for specific gut commensals, including a significant association between *Parabacteroides* and T1D before disease onset. However, they are descriptive and do not establish causality. *P. distasonis* is a gram-negative, strictly anaerobic gut commensal of humans and other animals. *P. distasonis* was previously reported for its beneficial effects in alleviating inflammatory arthritis (Sun *et al.* 2023), colitis (Cuffaro *et al.* 2020), type 2 diabetes (Liu *et al.* 2023), obesity (Cuffaro *et al.* 2023), nonalcoholic steatohepatitis (NASH) (Wei *et al.* 2023), chronic abdominal pain (Gervason *et al.* 2024), and tumorigenesis (Koh *et al.* 2020, Wang *et al.* 2024) in different mouse models. *P. distasonis* colonization also increased intestinal barrier integrity and modulated inflammatory markers in A/J mice (Koh *et al.* 2020).

In our previous study (Girdhar *et al.* 2022), we identified an insB9–23 mimic in the hypoxanthine phosphoribosyltransferase (hprt) protein of *Parabacteroides distasonis* (hprt4-18). Insulin B-chain amino acids 9–23 (insB9–23) (Daniel *et al.* 1995) is one of the most immunodominant T-cell epitopes in the islets (Pathiraja *et al.* 2015, Kent *et al.* 2017, Michels *et al.* 2017) and peripheral blood of human T1D patients (Alleva *et al.* 2001, Yang *et al.* 2014, Battle *et al.* 2015, Nakayama *et al.* 2015). The insB9–23 sequence is conserved between humans and NOD mice, a widely used model for T1D (Akbarpour *et al.* 2015, Russo *et al.* 2021). In the NOD mouse (Ferris *et al.* 2014, Unanue 2014), over 90% of the anti-insulin CD4^+^ T-cell clones target amino acids 9–23 of the insulin B chain (insB9–23) (Daniel *et al.* 1995). In our previous study, we hypothesized that T1D is triggered by a gut microbiota-derived epitope through a molecular mimicry mechanism, and we demonstrated that the hprt4-18 peptide can activate insB9–23-specific T cells. Furthermore, colonization of the female NOD mice with *P. distasonis* enhanced T1D onset, increasing inflammatory cells in the spleen and pancreatic lymph nodes (PLNs). Finally, using gut microbiome metagenome data from the DIABIMMUNE study, we showed that children harboring the hprt4-18 sequence had a higher rate of seroconversion.

Recent studies suggest that alterations in gut microbes and T1D could be linked to several factors, such as increased gut permeability (Monsted *et al.* 2021) and microbial metabolites, including SCFAs (Bell *et al.* 2022, Vatanen *et al.* 2024) and other proinflammatory metabolites. Here, we further investigated the impact of live and heat-inactivated (HI) *P. distasonis* on the host and diabetes onset. We focused on the gut microbiome, intestinal immune cell composition, gut permeability, circulating cytokine levels, and serum metabolome in specific pathogen-free (SPF) NOD mice and germ-free (GF) female NOD mice.

## Materials and methods

### Animals

NOD/ShiLtJ mice were purchased from the Jackson Laboratory facility. Mice were maintained and bred in the Boston College Animal Care Facility. The mice were housed in SPF conditions with unrestricted access to autoclaved water, food (Lab diet, #5008), and bedding, under a 12 h light:12 h darkness cycle. All the animal experiments were conducted as per the regulations and ethics guidelines of the National Institutes of Health (NIH) and were approved by the Institutional Animal Care and Use Committee (IACUC) of Boston College (Protocol No.#B2019-003, B2022-006, 2019-004, and 2022-010). Mice were weaned at 3 weeks of age.

NOD GF mice were housed in sterile conditions utilizing Trexler-type plastic isolators. Mice were exposed to a 12 h light:12 h darkness cycle and provided with autoclaved tap water and irradiated sterile pellets (breeding diet: Altromin 1414, Altromin, Germany) *ad libitum*. The sterility of NOD GF mice was verified biweekly by ensuring the absence of bacteria, molds, and yeast through aerobic and anaerobic cultivation of mouse feces and swabs from the isolators in meat-peptone broth, Sabouraud-dextrose, and VL (Viande-Levure), followed by plating on blood, VL, and Sabouraud agar plates. The animal experiments were conducted as per the regulations and ethics guidelines approved by the Committee for Protection and Use of Experimental Animals of the Institute of Microbiology of the Czech Academy of Sciences, v.v.i. (approval ID: 117/2013).

### Animal treatment

*P. distasonis* D13 strain was purchased from Dr Emma Allen-Vercoe’s laboratory at the University of Guelph. Dr Allen-Vercoe’s group isolated this bacterium from the colon of an ulcerative colitis patient. *P. distasonis* D13 was cultured anaerobically in Tryptic Soy Broth supplemented with vitamin K and hemin. Colony-forming units (CFU) were determined by plating serial dilutions on blood agar plates. For oral gavage, the bacteria were grown overnight, and the optical density was measured and adjusted based on CFU calculations to ensure accurate dosing. Three-week-old NOD mice, after weaning, were colonized with *P. distasonis* D13 as described previously (Girdhar *et al.* 2022). Briefly, the mice were orally gavaged for 4 weeks with either saline or live *P. distasonis* bacteria at a concentration of 1 × 10^8^ CFU/mouse/day. Bacterial colonization was determined at 10 weeks of age. Fecal DNA was extracted using the ZymoBIOMICS DNA Miniprep Kit (Zymo Research, USA) following the manufacturer’s instructions. qPCR was conducted using the QuantStudio 3 Real-Time PCR System (Applied Biosystems, USA) and Power SYBR Green Master Mix (Applied Biosystems) with the primer sequences: *P. distasonis* (Fw: TCG​AGT​TTT​TGC​CGG​CTT​TG; Rv: CGC​TTT​CAA​TCG​AGC​TTC​CG), Eubacteria (Fw: ACT​CCT​ACG​GGA​GGC​AGC​AGT; Rv: ATT​ACC​GCG​GCT​GCT​GGC). The relative abundance of *P. distasonis* was calculated by normalizing with Eubacteria. For GF mice colonization, GF mice were orally gavaged once either with saline or *P. distasonis* bacteria at the concentration of 1 × 10^8^ CFU/mouse. Bacterial colonization was determined at 10 weeks of age. Mice were sacrificed at 12 weeks of age, and organs such as the pancreas, intestine, and serum were collected to perform further analysis.

### Heat-inactivated *P. distasonis* (HI *P. distasonis*) preparations and oral gavage

For HI P. *distasonis* preparation, *P. distasonis D13* was cultured in Tryptic Soy Broth in an anaerobic chamber. *P. distasonis* was aliquoted at a concentration of 10^9^ CFU/100 μL in a microcentrifuge tube and was heated at 80°C for 45 min. HI was confirmed by plating on blood agar plates (Supplementary Fig. 4B (see section on Supplementary materials given at the end of the article)). Three-week-old female NOD mice were gavaged 5 days a week with HI P. *distasonis* for 4 weeks. qPCR was performed using *P. distasonis*-specific primers on fecal samples collected at 10 weeks of age. At 12 weeks of age, the mice were sacrificed to assess insulitis (*n* = 4–5 mice/group), and organs were harvested to determine the immune cell composition for further analysis as previously described (Girdhar *et al.* 2022) (Fig. 5B).

### Histopathological sectioning and staining

The formalin-fixed pancreas was dehydrated using an ethanol gradient, followed by embedding in paraffin to perform histological and eosin staining. The pancreas was sectioned transversely, and every other section (spaced 30 μm apart) was collected. Each block was trimmed to 30 μm and 5 μm sections using a Leica RM2155 microtome and was mounted onto slides. The paraffin sections were then stained with a hematoxylin and eosin (H&E) staining kit (Vector Laboratories, USA). Images of the islets were captured using a Zeiss AxioImager Z2 upright microscope to determine the insulitis score. The islets were scored blindly as follows: no insulitis, peri-insulitis, moderate insulitis, and severe insulitis. The insulitis scores were analyzed, and treatment groups were compared using an unpaired Student’s *t*-test with Welch’s correction or one-way ANOVA with post hoc test. The insulitis index was calculated to quantify the degree of inflammation and immune cell infiltration in the islets. No-insulitis islets (0% infiltration) were scored as 0, peri-insulitis islets (<25% infiltration) were scored as 1, moderate insulitis islets (<50% infiltration) were scored as 2, and severe insulitis islets (>50% infiltration) were scored as 3.

### Intraepithelial lymphocyte (IEL) isolation and flow cytometry

Intraepithelial lymphocyte (IEL) cells were isolated following previously established protocols (Girdhar *et al.* 2023). Briefly, small intestines were collected post-sacrifice, washed with phosphate-buffered saline (PBS), and longitudinally cut into 1-inch pieces to expose the inner epithelial layer. The intestine pieces were thoroughly washed with PBS containing 2% fetal bovine serum (FBS) to remove fecal particulates, repeating the process 2–3 times. Subsequently, the washed intestine pieces were agitated in freshly prepared 1 mM dithiothreitol (DTE) solution for 20 min. After incubation, cells were collected from the supernatant and filtered through a 70 μm filter. The DTE incubation process was repeated once more to ensure maximum cell recovery. The collected cells from both rounds of incubation were combined and further purified using a 44/67 Percoll gradient to isolate the intraepithelial lymphocyte (IEL) cells. The cell suspension was washed twice with RPMI media containing FBS before surface labeling with appropriate fluorochrome-conjugated monoclonal antibodies, as detailed in Supplementary Table S2. For tetramer staining, cells were first incubated with tetramers (1:200 dilution) for 30 min at room temperature, followed by staining for surface antigens. We obtained four I-A^g7^ MHC class II tetramers from the NIH Tetramer Core Facility. Two were custom tetramers: one loaded with a modified insulin B9–23 peptide (SHLVEALYLVAGERG, C19A) and labeled with PE, and the other with a modified *P. distasonis* HPRT4–18 mimic peptide (LVELLYLVASEYLNH, C19A) labeled with APC. As negative controls, we also requested two CLIP peptide-loaded I-A^g7^ tetramers (PVSKMRMATPLLMQA), labeled with PE and APC, respectively. The native insulin B9–23 peptide sequence (SHLVEALYLVCGERG) contains a cysteine residue that was incompatible with the peptide exchange protocol used by the facility. Therefore, the tetramer was generated using a modified sequence (SHLVEALYLVAGERG, C19A) recommended by the facility. In addition, two independent peptide synthesis companies were unable to generate the original *P. distasonis* peptide (RILVELLYLVCSEYL) due to its hydrophobicity. As a result, we used a modified version (LVELLYLVASEYLNH) to successfully synthesize the peptide for tetramer production. The flow cytometry panel was optimized using varying concentrations of antibodies. Isolated cells were counted using a hemocytometer and stained on ice for 15 min with an antibody cocktail as described previously in staining buffer (PBS supplemented with 2% FBS) (Girdhar *et al.* 2022). The cell suspension was then washed three times and fixed with 1% paraformaldehyde. The samples were analyzed using a BD FACSAria flow cytometer, and the acquired data were analyzed using FlowJo10 software. The gating strategy employed to identify the cell population is described in Supplementary Figs 2 and 3.

### 16S rRNA gene sequencing

Fecal pellets were obtained from 10-week-old colonized NOD mice, immediately snap frozen, and stored at −80°C. Stored fecal pellets were used to obtain DNA samples using a Qiagen PowerFecal Pro DNA isolation kit. 16S sequencing was performed at the TGen Integrated Microbiomics Center (TIMC). Bacterial DNA was quantified by the BactQuant assay (Liu *et al.* 2012). 16S rRNA gene libraries were created by amplifying the variable region 4 (V4) using dual-index primers that included the Illumina adapters (Kozich *et al.* 2013). To this end, we used the universal primer pair 515F/806R, which is the standard for amplifying the V4 hypervariable region of the bacterial 16S rRNA gene in microbial community profiling. The primer sequences were: 515F (forward), 5′-GTGCCAGCMGCCGCGGTAA-3′, and 806R (reverse), 5′-GGACTACHVGGGTWTCTAAT-3′. The quality of the 16S rRNA amplicon library pool was assured using TapeStation, Qubit, and KapaQuant analysis. Amplicons were pooled and sequenced on one Illumina MiSeq Nano (2 × 250 bp) run. The resulting data from the MiSeq run (MiSeq 1027) yielded 711,008 bp that successfully passed the filter, with a read range of 6,497–28,665 reads per sample. The average quality score (Q30) obtained was 32.9.

### Serum metabolomic analysis

We completed our experiments as previously described (Kiebish *et al.* 2020, Tsuji *et al.* 2024). All serum samples were thawed at room temperature on a rotating plate for 20–30 min and then kept on ice during aliquoting. Each sample was vortexed before extracting 75 μL with 450 μL of cold extraction solvent (3:3:2 IPA: I: H_2_O at −20°C). An additional 5 μL of serum from each sample was combined and extracted (1.44 mL cold extraction solvent) to form a pooled QC sample. Alongside the samples, five QC plasma aliquots (75 μL each) were extracted following the same protocol. All sample mixtures were vortexed for 5 s and stored overnight at −20°C. After the overnight extraction, all samples, QC plasma, and the pooled sample were centrifuged at 21,000 *g* for 10 min. The supernatant (100 μL) was then transferred to glass LC-MS vials for analysis. Samples were analyzed on a Sciex 5500 Triple Quadrupole Mass Spectrometer (Sciex, USA) coupled to a Shimadzu NEXERA XR UPLC system (Shimadzu, USA). A two-solvent liquid gradient system consisting of 50 mM ammonium bicarbonate at pH = 9.4 (aqueous) and 100% Acetonitrile (organic) was used over 30 min, applying hydrophobic interaction liquid chromatography (HILIC) to separate targeted metabolites. The binary gradient was as follows: starting at 85% B, hold until the 3 min mark, then linear decrease to 70% B from 3 to 11 min, decrease to 40% B from 11 to 14 min, decrease to 25% B from 14 to 22 min, decrease to 2% B from 22 to 23 min, hold at 2% B from 23 to 28 min, then increase back to 85% B from 28 to 29 min, and hold at 85% B until 29.3 min. The flow rate was constant at 0.3 mL/min, and the column compartment was 35°C. An ApHeraTM NH2 HPLC column (5 μm, 15 cm × 2 mm) coupled to an ApHeraTM NH2 HPLC guard column (5 μm, 1 cm × 2 mm) and a pre-column filter (0.5 μm A-102 frit) was used for chromatographic separation. In electrospray ionization, the curtain gas pressure was 35 psi, collision gas 8 psi, spray voltage 4.5 kV, and temperature 500°C. Samples were analyzed using a scheduled MRM method with a 4 min detection window for each metabolite transition. The resulting data were trimmed of non-detected metabolites and then statistically analyzed via MetaboAnalyst based on desired sample groupings, using mean normalization, log transformation, and Pareto scaling. The metabolite acquisition list is shown in Supplementary Table S5.

### Luminex assay

Luminex cytokine assay was performed utilizing the Mouse Cytokine/Chemokine Magnetic Bead Panel (Millipore Sigma, USA; # MCYTOMAG-70K). Reagents were prepared as per kit instructions (including wash buffers, sample matrix, beads, and standards). The assay was performed as per the manufacturer’s instructions. Briefly, 200 μL of Wash Buffer was added into each well, and the sealed plate was shaken for 10 min at room temperature. Following this, the Wash Buffer was decanted, 25 μL of each standard or control was added to the appropriate wells, and 25 μL of Assay Buffer was added to the sample wells. For background, standards, and control wells, 25 μL of the Serum Matrix solution was added. 25 μL of diluted serum samples (1:1 in assay buffer) were added into the appropriate wells. 25 μL of premixed beads were added to each well. The plate was sealed, wrapped with foil, and incubated with agitation overnight at 2–8°C. After incubation, the plate was washed twice, and 25 μL of detection antibodies were added into each well and incubated with shaking for 1 h. Next, 25 μL of Streptavidin-Phycoerythrin was added to each well containing detection antibodies; the plate was sealed, covered with foil, and incubated with shaking for 30 min. Following incubation, the plate was washed twice, and 150 μL of Sheath Fluid was added to all wells; then, the beads were resuspended on a plate shaker for 5 min. The plates were then read on Bio-Plex®200 following manufacturers’ specifications and using Bio-Plex Manager software v6.2. The samples were analyzed for 32 analytes, including Eotaxin, G-CSF, GM-CSF, IFN-γ, IL-1α, IL-1β, IL-2, IL-3, IL-4, IL-5, IL-6, IL-7, IL-9, IL-10, IL-12 (p40), IL-12 (p70), IL-13, IL-15, IL-17, IP-10, KC, LIF, LIX, MCP-1, M-CSF, MIG, MIP-1α, MIP-1β, MIP-2, RANTES, TNF-α, and VEGF.

### Bioinformatic analysis and statistics

Bacterial 16S rRNA amplicon sequencing reads were filtered and trimmed to assess microbial diversity and perform statistical analysis. Dada2 (Callahan *et al.* 2016) was used to convert amplicon sequences into an amplicon sequence variant (ASV) table using the ribosomal database project training set (Cole *et al.* 2014). The R (version 4.1.2) packages Phyloseq (McMurdie & Holmes 2013) and vegan were employed for exploratory and inferential analyses, including alpha and beta diversity estimates, non-metric multidimensional scaling (NMDS) analysis using Bray–Curtis dissimilarity, principal components analysis (PCA), and taxa agglomeration. Statistical significance for alpha diversity was assessed using ANOVA, while PERMANOVA was used for Bray-Curtis dissimilarity. Differential ASV abundance was evaluated at each time point using edgeR (Robinson *et al.* 2010) with two-sided empirical Bayes quasi-likelihood F-tests. *P*-values were corrected using the Benjamini–Hochberg false discovery rate (FDR), with FDR <0.05 considered statistically significant (Glickman *et al.* 2014).

### Statistics

Statistical analysis was conducted using the unpaired Student’s *t*-test to compare two groups. Significance levels were denoted as *P < 0.05, **P < 0.01, and ***P < 0.001. For insulitis scores, either an unpaired Student’s *t*-test or a one-way analysis of variance (ANOVA) with Tukey’s post-hoc test was performed to compare multiple groups. Significance was determined at *P* < 0.05. All statistical analyses were conducted using GraphPad Prism Version 9.0 unless otherwise specified in the figure legends.

## Results

### *P. distasonis* colonization has a limited impact on gut microbiome composition in female NOD mice

**Figure 1:**
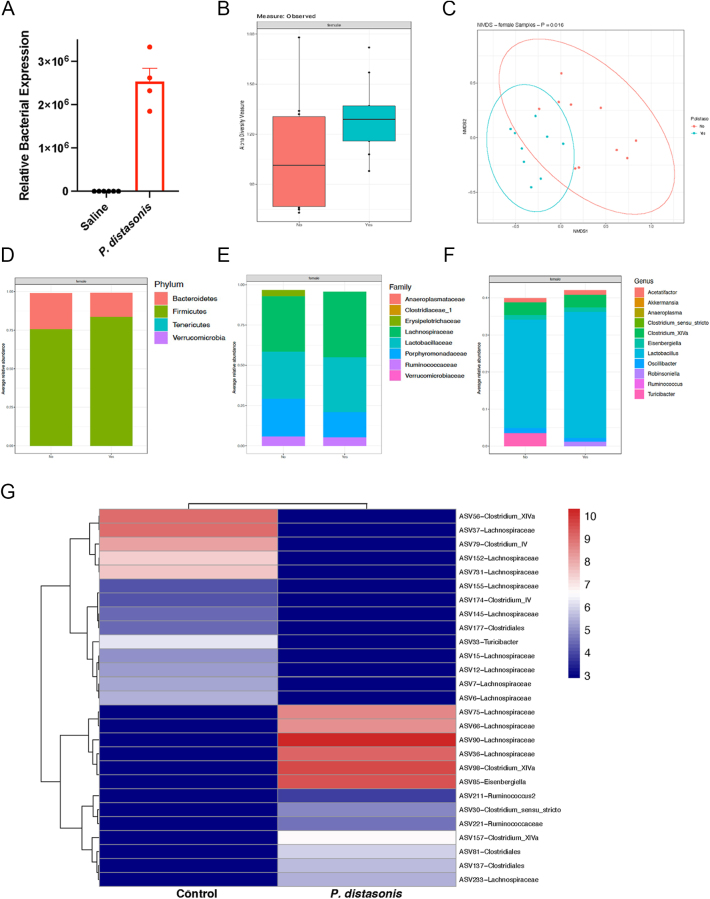
The effect of *P. distasonis* on the gut microbiome profile of NOD female mice. (A) Relative abundance of *P. distasonis* in (saline) control and *P. distasonis*-orally gavaged NOD female mice at 10 weeks of age (*n* = 4/group). Statistical analysis was performed using the two-tailed unpaired Student’s *t*-test. (B) Alpha diversity, (C) beta diversity, (D) average relative abundance of bacterial phylum, (E) family, and (F) genera. Statistical analysis was performed with the Benjamini and Hochberg method using two-tailed *t*-tests to control the FDR. (G) Heatmap showing the relative abundance of the ASVs significantly different between *P. distasonis*-colonized and saline-gavaged mice (*n* = 9–10/group). Each column represents the mean of each group, and each row represents an ASV.

To examine the impact of *P. distasonis*, we first focused on the gut microbiome composition after the colonization. To this end, we used fecal samples from 10-week-old female NOD mice, which were either orally gavaged with *P. distasonis* or saline for 4 weeks (starting with 3-week-old mice). *P. distasonis* colonization did not affect alpha diversity, indicating that overall species richness remained stable (Fig. 1A and Supplementary Fig. 1A, B, C). On the other hand, the beta diversity was altered (*P* = 0.016) (Fig. 1B). We also observed no significant differences at the phylum (Fig. 1C), family (Fig. 1D), class, and order levels (Supplementary Fig. 1D and E), as well as at the genus level (Fig. 1E). In total, we identified 188 amplicon sequencing variants (ASVs) (Supplementary Table S1), with 17 ASVs showing increased abundance and 11 ASVs showing decreased abundance following *P. distasonis* colonization (*P* < 0.05). Interestingly, most significant alterations within the annotated 28 ASVs occurred in members of the *Lachnospiraceae* family. However, the alteration was not unidirectional; while ASV6, ASV7, and ASV37 were decreased, ASV36, ASV85, and ASV66 were increased (Supplementary Table S1 and Fig. 1F). Overall, these results indicate that *P. distasonis* colonization had a limited impact on the gut microbiome composition.

### *P. distasonis* decreases inflammatory immune cells in the small intestine without altering the expression of gut permeability-related genes

We previously reported that *P. distasonis*-colonized NOD female mice had an increase in CD8+ T cells, F4/80+ macrophages, and dendritic cells, along with a decrease in FoxP3+ regulatory T cells in splenocytes. Similar alterations were also observed in the pancreatic lymph nodes for FoxP3+ T-regulatory cells and macrophages. However, the effect of *P. distasonis* on systemic inflammation and intestinal immune cell composition remained elusive. To determine whether the elevated incidence of T1D is associated with increased immune activation in the gut, we examined the direct effects of *P. distasonis* in the intestines, where the bacterium directly interacts with the immune system. We initially assessed the intestinal intraepithelial lymphocyte (IEL) composition in 12-week-old female NOD mice.

**Figure 2:**
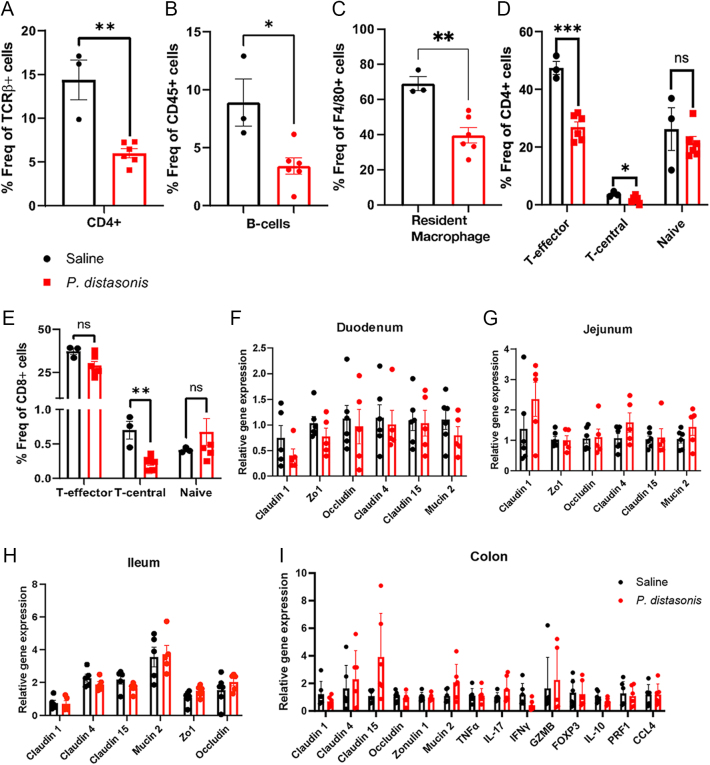
The effect of *P. distasonis* colonization on intestinal inflammation in NOD female mice. (A) CD4+ cells as a percentage of TCR-β+ CD45+ immune cell subsets. (B) Percentage of B-cells in CD45+ cell subsets. (C) Percentage of CD11b+ (dendritic cell subsets in CD45+ immune cells). (D) CD44^hi^CD62L^lo^ (T_EM_), CD44^hi^ CD62L^hi^ (T_CM_), CD44^lo^ CD62L^hi^ (naive) T-cells in CD4+ T-cells. (E) CD44^hi^CD62L^lo^ (T_EM_), CD44^hi^ CD62L^hi^ (T_CM_), CD44^lo^ CD62L^hi^ (naive) in CD8+ T-cell subsets population in saline- and *P. distasonis*-gavaged mice. Relative gene expression of gut permeability-related genes in (F) duodenum, (G) jejunum, and (H) ileum in *P. distasonis*-colonized NOD mice compared to saline NOD mice (*n* = 5/group). Data were expressed as mean ± SEM. *P < 0.05, **P < 0.01, ***P < 0.0001. Statistical analysis was performed using the two-tailed unpaired Student’s *t*-test.

Flow cytometry data revealed a 2.4-fold decrease in CD4+ T-cells (Fig. 2A) and a 2.6-fold decrease in B cells (Fig. 2B) in *P. distasonis*-colonized mice. However, there was no significant difference in total T cells, CD4+CD25+ T cells, and CD8+ T cells (Supplementary Fig. 2C, D, E). In addition, we examined the innate immune cell composition in IELs, including dendritic cells, macrophages (total, resident, and circulatory macrophages), and eosinophils. We observed a 1.7-fold decrease in resident macrophages (Fig. 2C) in *P. distasonis*-colonized mice, while no significant differences were observed in dendritic cells, total macrophages, circulatory macrophages, and eosinophil populations (Supplementary Fig. 3B, C, D, E, F). Further analysis of CD4+ and CD8+ T-cell subsets showed a 1.76-fold decrease in CD4+ T-effector and a 2.1-fold decrease in CD4+ T-central cell populations (Fig. 2D). We also observed a three-fold decrease in CD8+ T-central memory cell population upon *P. distasonis* colonization in IELs (Fig. 2E). Overall, *P. distasonis* colonization resulted in a reduction of CD4+ effector T cells, both CD4+ and CD8+ central memory T cells, B cells, and resident macrophage populations in the small intestine. These findings suggest that *P. distasonis* does not stimulate an increase in inflammatory immune cell populations upon colonization. Rather, colonization appears to promote a less inflammatory intestinal environment, though further studies are needed to fully characterize the mechanisms involved. To determine the impact of *P. distasonis* colonization on the systemic immune response, we employed a Luminex assay analyzing 32 different cytokines, chemokines, and growth factors on serum samples from 12-week-old *P. distasonis*-colonized female NOD mice compared to control mice (*n* = 8–9/group). From those 32 analytes, only IL-15 levels were 1.9-fold decreased upon *P. distasonis* colonization (Supplementary Fig. 4). Altogether, these results are consistent with our observations in the intestines and indicate that *P. distasonis* does not stimulate a nonspecific, pro-inflammatory, or anti-inflammatory cytokine response that can explain increased insulitis or diabetes rates of NOD female mice upon colonization. Subsequently, we investigated the impact of *P. distasonis* on gut permeability-related gene expression as another potential mechanism that might stimulate inflammation and contribute to diabetes acceleration. Gene expression analysis of claudin family proteins (claudin1, claudin4, and claudin15) and tight junction proteins (ZO1, occludin, and mucin2) in the duodenum, jejunum, ileum, and colon did not reveal significant changes upon *P. distasonis* colonization (Fig. 2F, G, H, I). Taken together, *P. distasonis* colonization does not increase expression of gut permeability-related genes in the small intestine.

### *P. distasonis* does not alter serum metabolome composition

**Figure 3:**
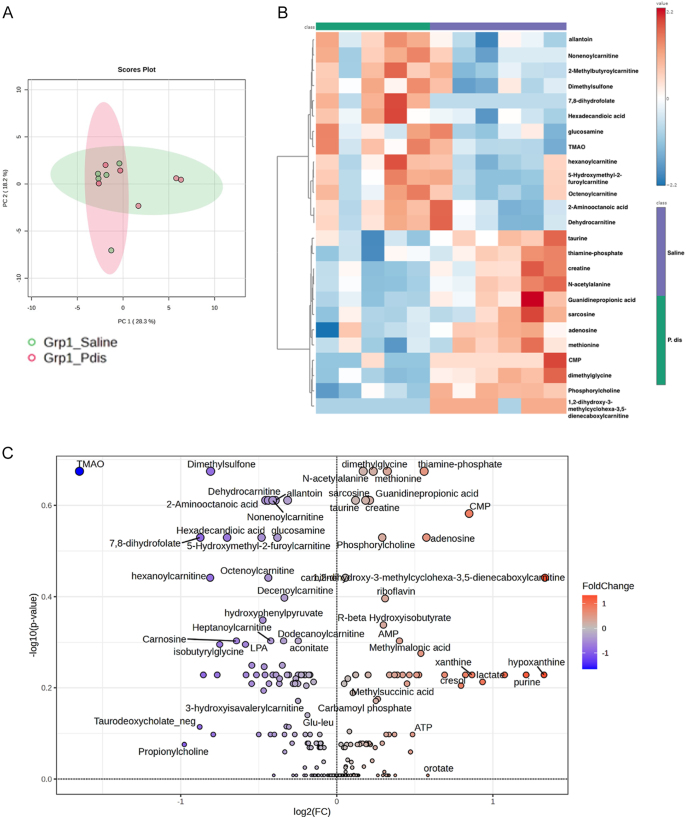
The effect of *P. distasonis* colonization on serum metabolite composition in NOD female mice. (A) PCA was performed using partial least squares-discriminant analysis (PLS-DA) of serum metabolites. (B) Heatmap showing 25 of the most altered metabolites between saline and *P. distasonis*-orally gavaged mice. Each column represents an individual mouse, and each row represents a metabolite. (C) Volcano plot of serum metabolites with fold change threshold (|log2 (FC)|>1.2) and *t*-test threshold (−log10(*P*) > 0.1). The red dots represent metabolites above the threshold. Fold changes are log2 transformed, and *P* values are log10 transformed for saline (*n* = 6) and *P. distasonis* (*n* = 5) orally gavaged NOD female mice.

To determine whether colonization has an impact on the serum metabolome, we employed targeted metabolomics using serum samples from 12-week-old female NOD mice. In total, 255 metabolites were determined in this analysis. The principal component analysis (PCA) did not reveal any significant differences between the groups (Fig. 3A). Among all 255 targeted metabolites, we identified no significant change in any of the metabolites between *P. distasonis* and the saline-treated group (Supplementary Table S3, Fig. 3B and C). Overall, these findings suggest that *P. distasonis* colonization has no impact on the serum metabolome composition.

### HI *P. distasonis* does not accelerate islet autoimmunity

**Figure 4:**
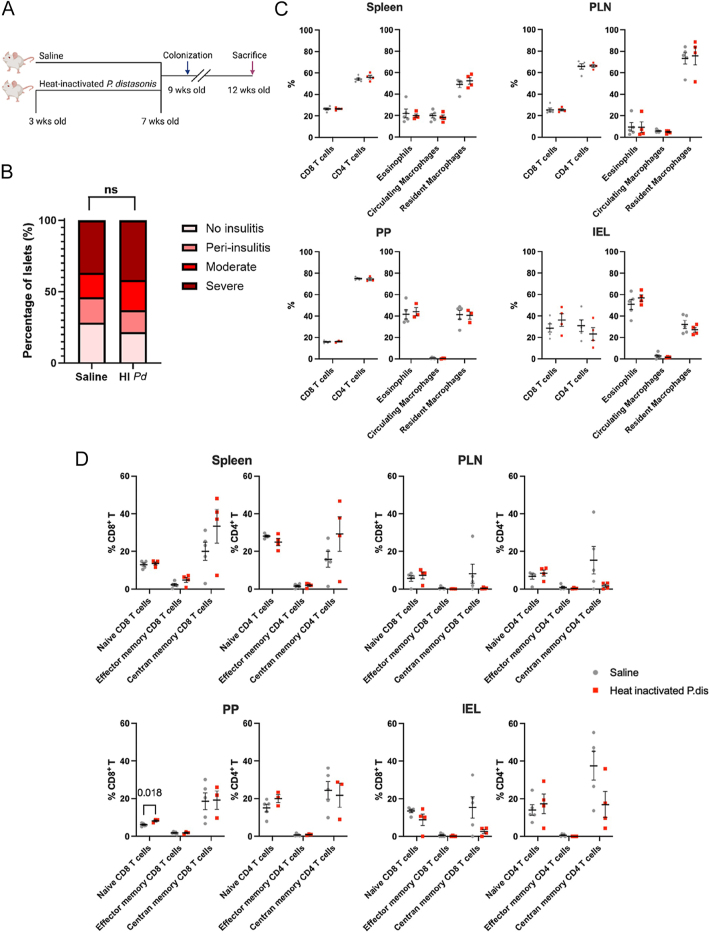
Heat-inactivated *P. distasonis* (HI *P. distasonis*) administration did not accelerate islet autoimmunity in NOD female mice. (A) Schematic overview of the HI *P. distasonis* oral gavage experiment. The blue arrow indicates the time point (week 9) when fecal samples were collected to test for *P. distasonis* colonization using qPCR. The red arrow indicates week 12, when the mice were sacrificed for pancreas collection to analyze insulitis. (B) Insulitis scores in saline- or HI *P. distasonis*-treated mice at 12 weeks of age (*n* = 5 saline, 4 HI *P. distasonis* female NOD mice) (*n* = 163–176 islets/group). (C) Percentage of immune cells (CD4+ T cells, CD8+ T cells, eosinophils, and macrophages) in the indicated organs. (D) Percentage of naïve (CD44^lo^ CD62L^+^), T_EM_ (effector memory; CD44^hi^ CD62L^lo^), and T_CM_ (central memory; CD44^hi^ CD62L^+^) in CD4^+^ and CD8^+^ T cells in the indicated organs. Statistical analysis was performed using the two-tailed unpaired Student’s *t*-test.

**Figure 5:**
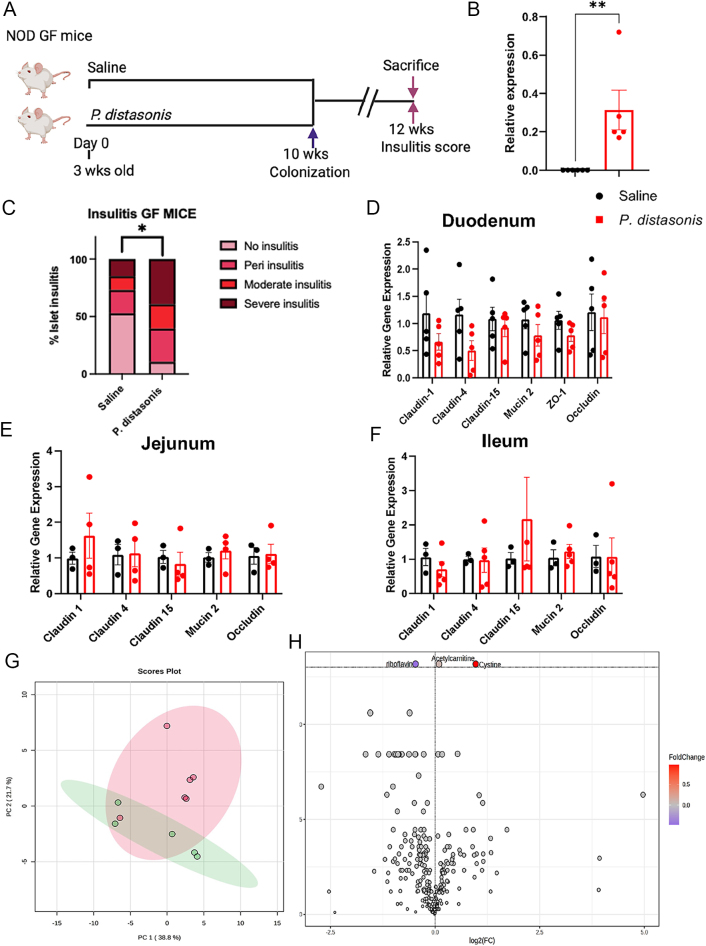
The effect of *P. distasonis* colonization on insulitis, gut permeability, and serum metabolome in GF NOD female mice. (A) Schematic overview of the *P. distasonis* oral gavage experiments (*n* = 5 saline, *n* = 6 *P. distasonis*). The time point of week 10 represents the fecal sample collection for the qPCR, the colonization experiment for *P. distasonis* colonization, and the red arrow shows the time point (week 12) for pancreata collection for insulitis analysis (*n* = 5 mice/group). (B) Relative abundance of *P. distasonis* in control and orally gavaged mice. (C) Quantification of insulitis scores from *P. distasonis*-colonized or saline-gavaged female NOD GF mice at week 12 (*n* = 5/group, *n* = 150–180 islets/group). Relative gene expression of gut permeability-related genes in duodenum (D), jejunum (E), and ileum (F) in *P. distasonis*-colonized NOD GF female mice compared to saline NOD GF mice (*n* = 4–5/group). Data were expressed as mean ± SEM. *p1.2 and *t*-tests threshold (−log10(*P*) > 0.1). The red dots represent metabolites above the threshold. Fold changes are log2 transformed, and *P* values are log10 transformed of saline (*n* = 5) and *P. distasonis* (*n* = 6) oral gavaged NOD GF female mice. Data were expressed as mean ± SEM. *P < 0.05, **P < 0.01,***P < 0.0001. Statistical analysis was performed using an unpaired Student’s *t*-test for insulitis and gene expression analysis. (G) PCA plot showing a comparison of serum metabolites between saline and *P. distasonis* oral-gavaged GF female NOD mice. (G) Heatmap showing 25 of the most altered metabolites between saline and *P. distasonis* oral-gavaged GF female NOD mice. Each column represents an individual mouse, and each row represents a metabolite. (H) Volcano plot of serum metabolites with fold change threshold (|log2 (FC)|>1.2) and *t*-tests threshold (−log10(*P*) > 0.1). The red dots represent metabolites above the threshold. Fold changes are log2 transformed, and *P* values are log10 transformed for saline (*n* = 5) and *P. distasonis* (*n* = 6) orally gavaged NOD GF female mice.

To determine the impact of *P. distasonis* colonization in the intestine, female NOD mice were orally gavaged with HI *P. distasonis* or saline for 4 weeks. No colonization of *P. distasonis* was observed in mice gavaged with HI *P. distasonis* (Supplementary Fig. 4C). While two of six mice in the HI *P. distasonis* group developed diabetes before analysis (Fig. 5E), the insulitis score was comparable between the remaining mice gavaged with HI *P. distasonis* and saline-treated control mice at 12 weeks (Fig. 4B). The composition of immune cells (CD4^+^ T cells, CD8^+^ T cells, eosinophils, and macrophages) and the phenotype of CD4^+^ or CD8^+^ T cells, defined by CD44 (a marker of activation and memory) and CD62L (a marker of lymphoid homing and differentiation status), were comparable between the groups, except for naïve CD8^+^ T cells in Peyer’s patches (Fig. 4 and Supplementary Fig. 5).

We generated two I-A^g7^ tetramers, one loaded with the *P. distasonis*-derived hprt4-18 peptide (LVELLYLVASEYLNH, C19A) and the other with the insulin B9–23 peptide (SHLVEALYLVAGERG, C19A), to evaluate whether colonization affects the abundance of antigen-specific CD4+ T cells. To validate tetramer specificity, we first tested them on insulin B9–23-specific IIT-3 T-cell hybridomas. As expected, the insulin B9–23 tetramer robustly stained IIT-3 cells, while the hprt4-18-loaded tetramer did not (Supplementary Fig. 4D), confirming that it does not cross-react with insulin-specific CD4+ T cells. Next, we assessed whether colonization with live or heat-inactivated *P. distasonis* (HI *P. distasonis*) influences insulin B9–23-specific CD4+ T cells *in vivo*. Female NOD mice were orally gavaged with saline, live *P. distasonis*, or HI *P. distasonis*, and the frequency of insulin B9–23 tetramer-positive CD4+ T cells was measured in the pancreatic and mesenteric lymph nodes at 10 weeks of age. No significant differences were observed among groups, suggesting that *P. distasonis* colonization does not promote the expansion of autoreactive CD4+ T cells targeting the insulin B9–23 epitope at this stage of disease (Supplementary Fig. 4E). Furthermore, it did not affect T-cell composition in the PLN, MLN, or spleen.

### *P. distasonis* accelerates insulitis in NOD GF female mice

To determine the isolated impact of *P. distasonis* colonization on insulitis and mitigate the potential effect of other gut microbes in SPF NOD mice, we utilized female GF NOD mice. The aim of using GF mice is to eliminate any confounding factors stemming from other gut microbes. The GF female NOD mice (*n* = 5–6) were orally gavaged once with *P. distasonis* after weaning (Fig. 5A). Colonization was confirmed in 10-week-old GF NOD mice using qPCR (Fig. 5B). The mice were sacrificed at 12 weeks of age to determine the insulitis scores. Consistent with our previous study in SPF NOD mice, *P. distasonis* colonization alone increased insulitis in GF NOD mice (Fig. 5C). It caused a 2.8-fold increase in severe insulitis in islets and a 5.6-fold decrease in islets with no insulitis compared to the control animals. These data indicate that *P. distasonis*, independent of other gut microbes, can induce insulitis in NOD mice. To investigate the mechanism of *P. distasonis*-induced insulitis and its potential link to gut permeability, we assessed the expression of gut permeability-associated genes. We assessed the gene expression of claudin1, claudin4, ZO1, claudin15, mucin2, and occludin in different parts of the small intestine. Similar to our findings in SPF mice, we did not observe any differences in the duodenum (Fig. 5D), jejunum (Fig. 5E), and ileum (Fig. 5F), indicating that *P. distasonis* colonization does not alter expression of gut permeability genes.

### *P. distasonis* colonization does not alter serum metabolite composition in GF NOD mice

To determine the direct impact of *P. distasonis* on the metabolome, we performed targeted metabolite analysis using serum samples obtained from the GF mice. In total, we identified 254 metabolites (Supplementary Table S4), and among them, only three metabolites were significantly altered. However, the magnitude of these changes was relatively low. The PCA did not identify significant differences between groups (Fig. 5G). Overall, our findings are consistent with our SPF findings and indicate that *P. distasonis* has a minimal impact on serum metabolome composition in GF mice (Fig. 5H).

## Discussion

T1D is one of the oldest chronic autoimmune diseases, and it is still not curable. Although we have better tools to manage the disease, it still decreases life expectancy by an average of 9.9 years (Arffman *et al.* 2023). We cannot prevent new cases because the trigger of T1D autoimmunity is unknown. Therefore, there is an urgent need to identify environmental factors contributing to T1D onset (Stewart *et al.* 2018). In this study, we examined the involvement of *P. distasonis* in T1D pathogenesis. As previously mentioned, the TEDDY and INNODIA studies reported that *Parabacteroides* was the most significantly associated genus with T1D onset (Houghton *et al.* 2018).

In our previous study, we also showed that the *P. distasonis* D13 strain colonization accelerates T1D onset in female NOD mice. To further define the impact of *P. distasonis* colonization on the host, we completed this comprehensive study. Our results on gut microbiome composition indicated a significant change in beta diversity, with 28 differentially abundant ASVs, mostly members of the *Lachnospiraceae* family. When we evaluated the effects of colonization on immune cell composition in the IELs, we observed a decrease in resident macrophages, B-cells, and CD4+ cells. Notably, there was also a reduction in CD4+ T-effector, T-central subsets, and CD8+ T-central subsets. Intestinal CD4+ T cells are a major population in IELs, crucial for maintaining host protective and homeostatic responses to gut microbes. However, an accumulation of CD4+ T cells, particularly the CD4+ T-effector subtype, is a hallmark of inflammation and inflammatory bowel disease (IBD) (Alleva *et al.* 2001, Shale *et al.* 2013, Brucklacher-Waldert *et al.* 2014). Similarly, B-cells play a significant role in increasing gut inflammation and villous atrophy in celiac disease (Brucklacher-Waldert *et al.* 2014, Lejeune *et al.* 2021). Reduction in these immune cells on colonization indicates a potential, local non-inflammatory phenotype.

*P. distasonis* significantly reduced intestinal inflammation in murine models of acute and chronic colitis (Gaifem *et al.* 2024) induced by dextran sulfate sodium (DSS) in BALB/c mice. This anti-inflammatory effect is mediated by a decrease in proinflammatory cytokines and stabilization of the intestinal microbiota (Kverka *et al.* 2011). In our study, we also observed a decrease in serum IL-15 concentration, consistent with our observations in the IELs. One of the potential mechanisms explaining this anti-inflammatory function is related to having a unique surface layer that breaks down complex polysaccharides and helps them blend with intestinal tissue (Fletcher *et al.* 2007). These findings on reduced intestinal inflammation align well with our results. Interestingly, treating high-fat diet-fed and ob/ob mice, two different models of type 2 diabetes and obesity, with *P. distasonis* CGMCC1.30169 reduced weight gain, hyperglycemia, and hepatic steatosis by activating intestinal gluconeogenesis and FXR pathways (Wang *et al.* 2019). The authors explained this phenotype with succinate production by the bacterium. However, we did not identify an increase in succinate in our SPF NOD and GF NOD mouse models.

There are also controversial findings indicating potential pathogenic effects of the bacterium. For example, the stools of Crohn’s disease patients repeatedly contained *P. distasonis* bacteria (Lopetuso *et al.* 2018). Likewise, in a DSS-induced colitis model, there was a correlation between *P. distasonis* abundance and the severity of colitis (Okayasu *et al.* 1990). *P. distasonis* inoculation increased inflammation in mice that already had Crohn’s disease (Ezeji *et al.* 2021). In a similar study, *P. distasonis* weakened the gut barrier and triggered inflammation, suggesting a link to IBD (Gonzalez-Paez *et al.* 2019). In addition, *P. distasonis* produces an enzyme that can inactivate antimicrobial peptides, including β-defensin 2, keratin-derived antimicrobial peptides (KAMPs), and human neutrophil peptide 3 (Xu *et al.* 2018). The differences observed in all these studies may be attributed to the variety of disease models, strains, and animal models tested.

Specifically, our results demonstrate that live *P. distasonis* exacerbates T1D, likely due to molecular mimicry mechanisms that activate autoreactive T-cells, while HI *P. distasonis* has no significant effect on immune cell composition. These findings are consistent with the broader concept that the immunological impact of gut bacteria depends on their viability, route of exposure, and the presence of specific immunomodulatory components. For example, studies on *Bacteroides fragilis* (BF), a related member of the *Bacteroidetes* family, have shown that its effects on T1D are highly context dependent. While oral administration of heat-killed BF can enhance immune regulation and delay T1D onset, systemic exposure to the same bacteria accelerates disease progression, highlighting the critical role of exposure route and microbial viability in determining immune outcomes. Importantly, the protective effects of BF are mediated by Polysaccharide A (PSA), as PSA-deficient strains fail to modulate disease progression (Sofi *et al.* 2019). Although our study did not examine systemic exposure to *P. distasonis*, the lack of effect observed with HI *P. distasonis* suggests that its immunomodulatory potential may also depend on specific molecular components that are inactivated by heat treatment.

Overall, our data indicate that *P. distasonis* colonization does not create a pro-inflammatory environment in the intestine of NOD mice. In contrast, it causes a reduction in the immune cells, indicating a potential anti-inflammatory effect in the gut. The underlying mechanism behind the decrease or migration of the immune cells from the intestine still needs to be determined. Notably, our results suggest that *P. distasonis* does not induce nonspecific inflammation but rather engages in a targeted immune interaction that contributes to T1D progression through molecular mimicry.

The molecular mimicry mechanism is based on the degeneracy of T-cell recognition (Mason 1998) and can be either pathogenic (Wucherpfennig & Strominger 1995) or protective (Mana *et al.* 2004). While molecular mimicry has long been postulated as a potential factor in autoimmune diseases (Harkiolaki *et al.* 2009, Greiling *et al.* 2018, Gil-Cruz *et al.* 2019), including T1D (Atkinson *et al.* 1994, Tian *et al.* 1994, Honeyman *et al.* 2010) (coxsackie B and rubella viruses), progress was hindered due to a lack of genomic sequences of the potential microbial proteins that might trigger this response. In our previous study, we took advantage of the growing genome databases for microbes, including growing microbiome datasets, and identified hprt4-18 (Girdhar *et al.* 2022). We showed its potential role in T1D pathophysiology, establishing cross-reactivity and determining the enhanced T1D in *P. distasonis*-colonized NOD mice. In this study, we comprehensively examined the effects on the host, ranging from intestinal immune cell composition to circulating metabolites. However, we could not identify additional mechanisms that might explain the increased T1D, thus supporting our original hypothesis. However, the specific role of hprt4-18 still needs to be determined in future studies using mutation models. The lumen presents a highly challenging environment for microbial survival (Yang *et al.* 2016, Chiang *et al.* 2024). Despite the evolutionary adaptations of gut microbes to colonize the gut, cell death and cell lysis remain inevitable. In addition to our findings, molecular mimicry was recently linked to other autoimmune diseases, including multiple sclerosis (MS). Recent studies identified pathogenic and cross-reactive antibodies as a potential link between Epstein–Barr virus (EBV) and MS onset. Researchers showed the cross-reactivity between EBV nuclear antigen EBNA1 and glial cell adhesion protein in the central nervous system (Lanz *et al.* 2022).

We acknowledge that our current tetramer approach does not demonstrate functional activation of insB9–23 population in response to *P. distasonis* colonization. To more rigorously interrogate T-cell activation dynamics, future studies will track endogenous CD8+ T-cells specific for the native ins15–23 epitope by designing specific peptide-MHC tetramers, comparing their expansion and phenotype to islet-specific glucose-6-phosphatase catalytic subunit-related protein (IGRP)-specific T-cells. Second, leveraging TCR transgenic mice with ins15–23-reactive T-cells to directly assess whether mimic peptide exposure drives clonal expansion, effector differentiation, or pancreatic infiltration. These models would provide definitive mechanistic insight into cross-reactive T-cell activation and help resolve whether molecular mimicry functionally contributes to β-cell autoimmunity in this context. While further studies are needed to establish a causal link between human T1D and *P. distasonis*, we believe that other pathobionts in the gut may also generate insB9–23-specific T-cells. Furthermore, hundreds of epitopes have been identified in the pathogenesis of T1D (Kent *et al.* 2017, James *et al.* 2020), with the potential for T-cells to be stimulated by several gut microbiota-derived mimic epitopes. Identifying these commensal microbes could provide a better understanding of gut–immune interactions and reveal mechanisms underlying autoimmune diseases such as T1D. Identification of causal factors will guide us in developing novel therapeutic tools to prevent, cure, and manage the disease.

## Supplementary materials













## Declaration of interest

Juan Henao and Michael Kiebish are employees of BPGbio, Inc.; however, they do not have any financial interest in T1D research or *P. distasonis*.

## Funding

TH and DŠ acknowledge support from Talking Microbes: Understanding Microbial Interactions within a One Health Framework (CZ.02.01.01/00/22_008/0004597). This work was also supported by the Beatson Foundation (Grant 2023-003 to E.A.) and a Diabetes Research Connection grant awarded to KG.

## Author contribution statement

KG and EA designed the research, analyzed the data, and wrote the paper. EA oversaw the project. KG, CH, AP, BJ, and LR assisted with animal experiments. DD assisted with 16S data bioinformatics analysis. KG, KM, and AR performed the cell isolations, FACS staining, and analysis, and BJ assisted with the HI *P. distasonis* experiment. MS, DS, UKG, and TH assisted with GF mice experiments and maintenance. MK and JH conducted the serum metabolomic analysis.
