# Supplementary material for: Impact of Parabacteroides distasonis colonization on host microbiome, metabolome, immunity, and diabetes onset
Source: J Mol Endocrinol. 2025 Aug 28;75(2):e250025. doi: 10.1530/JME-25-0025 (PMC12400532; doi:10.1530/JME-25-0025)

Supplementary Figure 1: Alpha diversity indexes and Relative abundance of gut bacterium upon *P. distasonis* colonization.

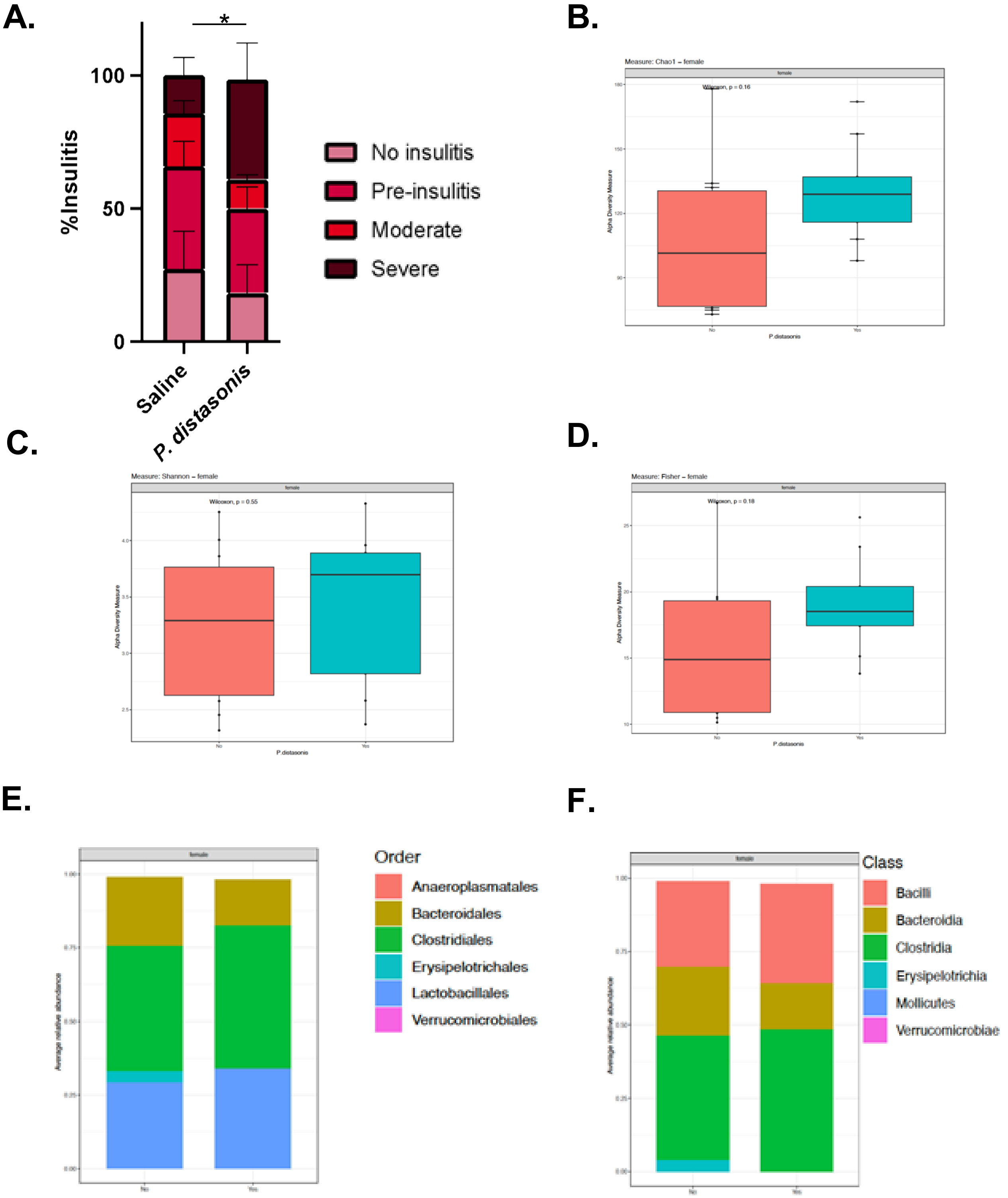

Supplementary Figure 2 : Gating Strategy and T-Cell Population in IEL of female NOD mice.

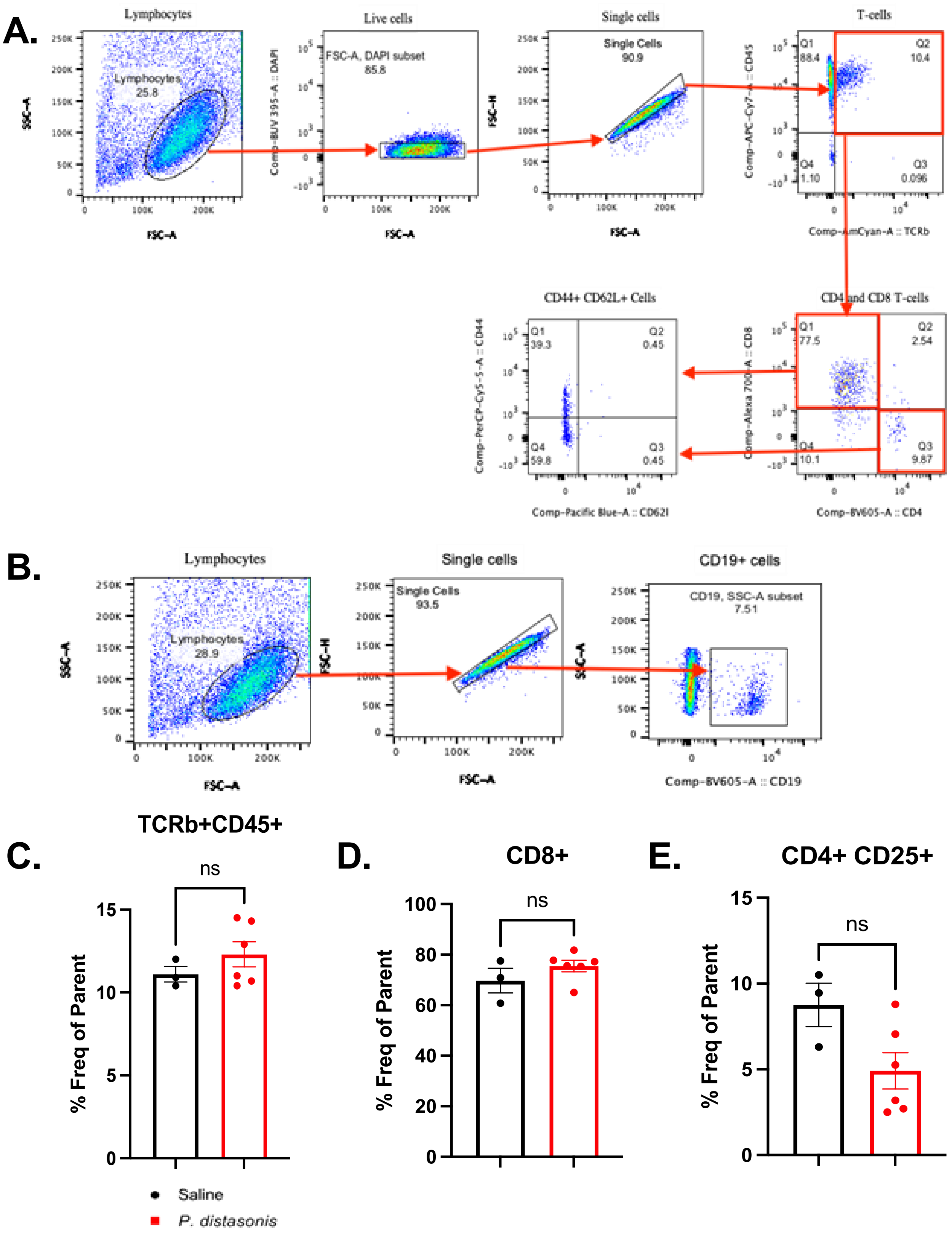

Supplementary Figure 3: Gating Strategy and Innate Cell Population in IEL of female NOD mice.

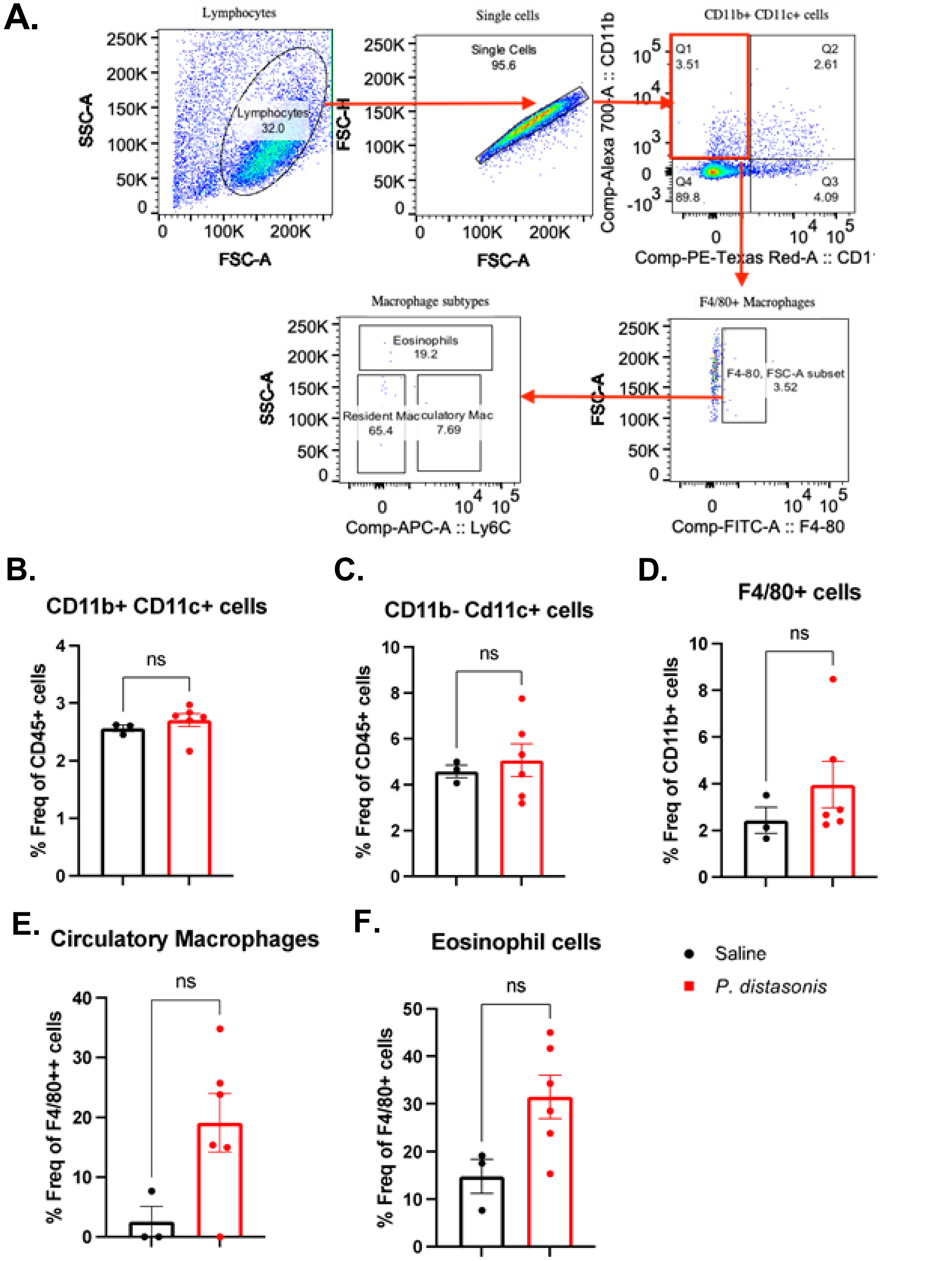

Supplementary Figure 4:

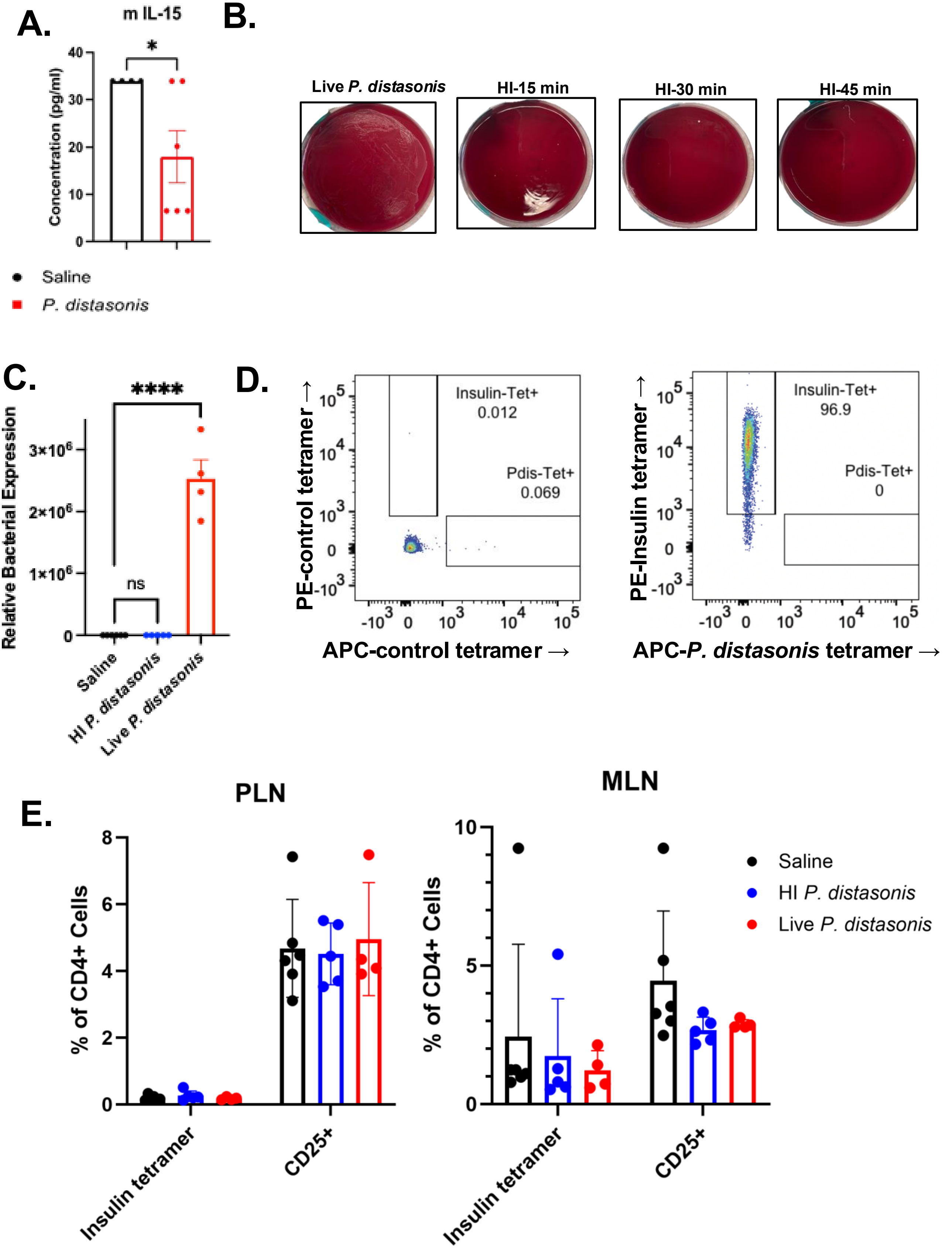

Supplementary Figure 5:

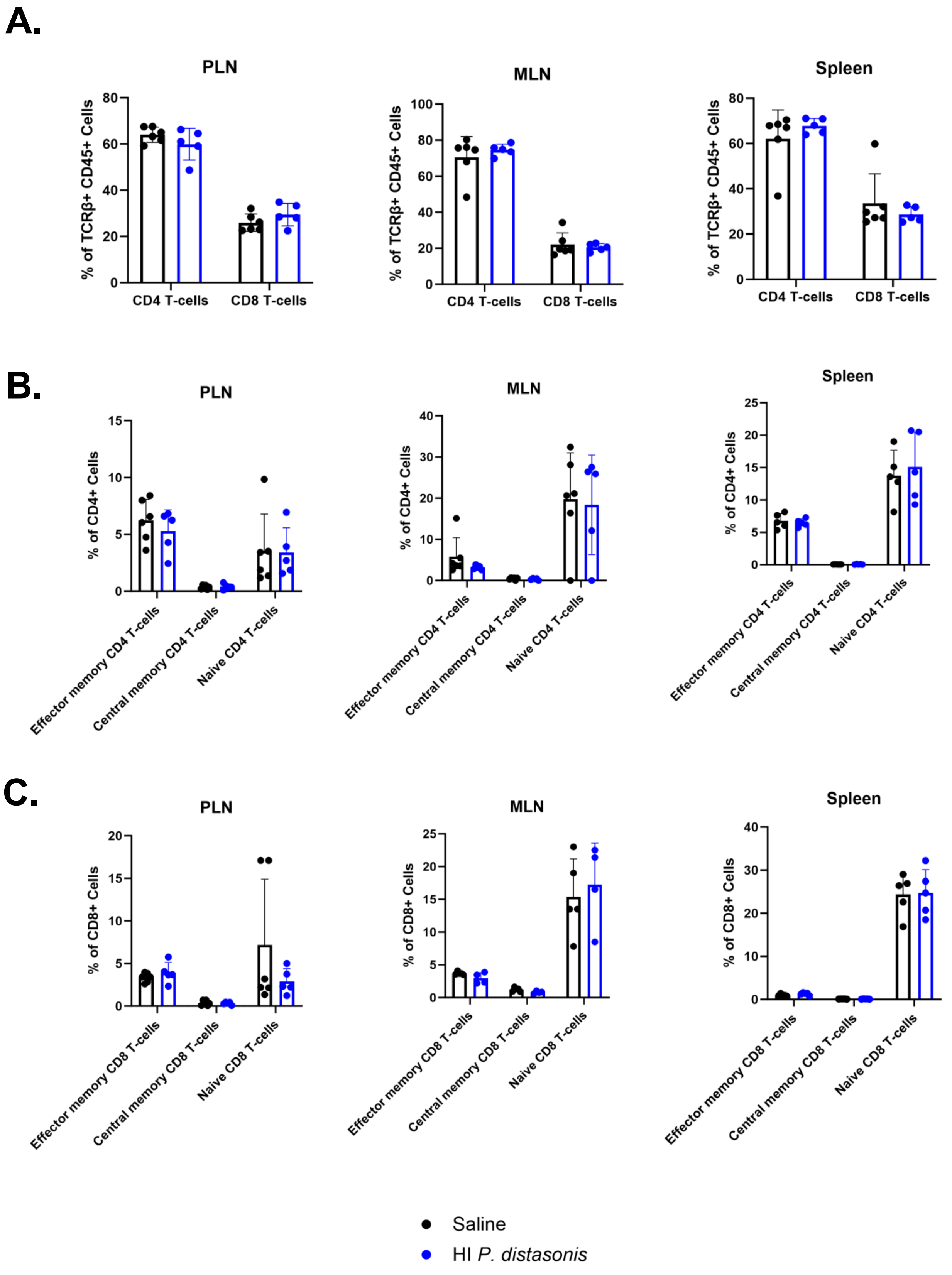

Supplement: Supplementary file 1 [file supplementary_figures.pdf]
